# Evolution of Nipah Virus Infection: Past, Present, and Future Considerations

**DOI:** 10.3390/tropicalmed6010024

**Published:** 2021-02-14

**Authors:** Naomi Hauser, Alexis C. Gushiken, Shivakumar Narayanan, Shyam Kottilil, Joel V. Chua

**Affiliations:** 1 Division of Infectious Diseases, University of California Davis Medical Center, Sacramento, CA 95817, USA; nehauser@ucdavis.edu; 2 Institute of Human Virology, University of Maryland School of Medicine, Baltimore, MD 21201, USA; agushiken@som.umaryland.edu (A.C.G.); snarayanan@ihv.umaryland.edu (S.N.); skottilil@ihv.umaryland.edu (S.K.)

**Keywords:** Nipah virus, Nipah virus infection, zoonoses, emerging infection, henipaviruses

## Abstract

Nipah virus (NiV) is a zoonotic paramyxovirus of the *Henipavirus* genus first identified in Malaysia in 1998. Henipaviruses have bat reservoir hosts and have been isolated from fruit bats found across Oceania, Asia, and Africa. Bat-to-human transmission is thought to be the primary mode of human NiV infection, although multiple intermediate hosts are described. Human infections with NiV were originally described as a syndrome of fever and rapid neurological decline following contact with swine. More recent outbreaks describe a syndrome with prominent respiratory symptoms and human-to-human transmission. Nearly annual outbreaks have been described since 1998 with case fatality rates reaching greater than 90%. The ubiquitous nature of the reservoir host, increasing deforestation, multiple mode of transmission, high case fatality rate, and lack of effective therapy or vaccines make NiV’s pandemic potential increasingly significant. Here we review the epidemiology and microbiology of NiV as well as the therapeutic agents and vaccines in development.

## 1. Introduction

Nipah virus (NiV) is a member of the *Henipavirus* genus of the *Paramyxoviridae* family and is a zoonotic virus with a high case fatality rate [1]. Our knowledge of the geographic distribution of NiV and the disease it causes, mode of pathogen transmission, and clinical manifestations of infection, have evolved over time. The first recognized human infection was in the Malaysian village of Kampung Sungai Nipah in 1998, initiating a deadly outbreak that lasted through 1999 [1,2]. Smaller sporadic outbreaks have since recurred nearly annually within South Asia with case fatality rates reaching greater than 90% [3,4].

The original human NiV infections were found to be associated with contact with swine, and it was later confirmed that NiV could be isolated from the nose and oropharynx of pigs [1,2,5,6]. Human infections were characterized by fever for up to 14 days, meningitis and/or encephalitis, with rapid neurological decline and progression to coma within 24 to 48 h [5]. Later outbreaks outside the Malay peninsula have been characterized by different transmission dynamics and clinical presentation, including the development of severe respiratory symptoms in addition to neurological complications, with human infection traced to the consumption of horse meat, proximity to other infected humans, and ingestion of raw date palm sap contaminated with the bodily fluids of bats [3,7,8,9,10,11,12,13,14].

Similar to lyssaviruses, filoviruses, coronaviruses, and the related Hendra virus, NiV is naturally hosted by pteropid bats [15,16]. Fruit bats found across Oceania, South and Southeast Asia, and sub-Saharan Africa are the natural reservoirs of NiV, and almost yearly outbreaks of NiV infections continue to occur throughout South and Southeast Asia [16,17]. The high case fatality rate associated with NiV infection, ubiquitous nature of the reservoir host, increasing deforestation, and expanding modes of transmission coupled with a lack of rapid diagnostics and effective vaccine or therapeutic agents make Nipah virus’ pandemic potential increasingly relevant. The following is a review of the epidemiology and microbiology of NiV as well as the drugs and vaccines being evaluated for the treatment and prevention of NiV infection.

## 2. Epidemiology

The original NiV infection cluster was first identified in September 1998 in Perak, Malaysia, followed by second and third clusters in the state of Negri Sembilan, with cases occurring primarily among adult men in contact with swine [1,2,5]. In March 1999, a cluster of 11 similar cases was recognized in Singapore among abattoir workers in contact with pigs imported from the outbreak regions of Malaysia, with NiV isolated from both affected patients and the pigs [1,2,6,17]. Thereafter, outbreaks continued to spread throughout Malaysia, leading to restrictions on swine imports to nearby Singapore, nationwide NiV testing among pigs in Malaysia, and ultimately the mass culling of over one million pigs from any farm in Malaysia with a confirmed infection. Together, these interventions ultimately resulted in the end of the outbreak nearly two years after initial discovery [1,2,5].

**Figure 1:**
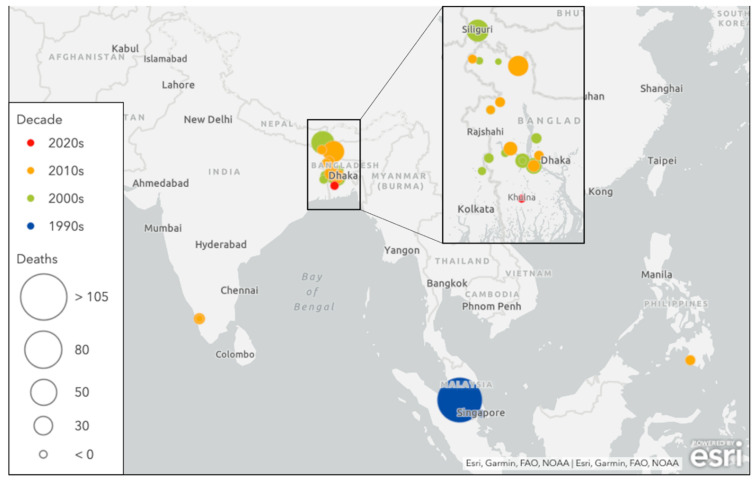
Nipah virus infection outbreak by decade. Illustration created by authors using Esri.

Sporadic NiV outbreaks have occurred in multiple countries throughout South and Southeast Asia since it was first discovered (Figure 1), and these recurrent episodes differ from the large early outbreaks of the Malayan peninsula with regards to clinical presentation, case fatality rate, and mode of transmission (Table 1).

**Table 1:** Nipah virus outbreaks listed in chronologic order with case fatality rates, exposure history, and clinical features. Legend—ARDS: acute respiratory distress syndrome; CFR: case fatality rate; CSF: cerebrospinal fluid; MODS: multiorgan dysfunction syndrome.

Country	Year(s)	Cases N	Fatalities (% CFR)	Exposure History, Transmission, Clinical Features	Reference
Malaysia	Sep 1998–Dec 1999	265	105 (38.5%)	Close contact with pigs (i.e., pig farmers) Preceded by symptomatic infection in pigs Febrile encephalitis Incubation period: 4 days to 2 months (90% in ≤ 2 weeks)	Chua 2000 [1] Goh et al., 2000 [18]
Singapore	March 1999	11	1 (9.1%)	Abattoir workers Pigs imported from a Malaysian farm affected by the virus Encephalitis and atypical pneumonia Nipah virus positive in CSF and tissue	Paton et al., 1999 [6]
Bangladesh	Jan 2001–Feb 2015 (17 outbreaks)	261	19 (75.9%)	Almost annual outbreaks since 2001 Direct consumption of fruit bat-contaminated date palm sap Human-to-human transmission (including nosocomial) More severe and rapid (ARDS, respiratory failure, MODS)	Luby et al., 2006 [13] Gurley et al., 2017 [10] Nikolay et al., 2019 [3]
India	Jan–Feb 2001 Apr 2007 May 2018 June 2019	92	68 (73.9%)	Four outbreaks (Siliguri 2001, Nadia 2007, Kerala 2018, 2019) Fever with acute respiratory distress ± neurologic symptoms Kerala 2018 outbreak most deadly (N = 19, CFR 91%). All except 1 due to nosocomial transmission	Chadha et al., 2006 [7] Banerjee et al., 2019 [19] Arunkumar et al., 2019 [20]
Philippines	Mar–Apr 2014	17	9 (52.9%)	Horse slaughtering and horse meat consumption Recent horse deaths reported Two healthcare workers who cared for patients Four cats and one dog that ate horse meat also died 11 with acute encephalitis syndrome, 5 influenza-like illness, and 1 meningitis Median incubation period: 8 days	Ching et al., 2015 [8]

A new and distinct strain of NiV, with infection characterized largely by acute severe respiratory symptoms, arose in Bangladesh and India in 2000–2001. Epidemiological studies revealed clustering around household members and hospital contacts without clear animal exposure, raising suspicion for human-to-human transmission [7,9,11].

Contact and exposure tracing also began to point toward the consumption of raw date juice as another common behavior among infected humans, emphasized in 2013 when two individuals in Dhaka, Bangladesh developed NiV infection after consuming raw date palm juice [10,12,13].

At the time NiV was first identified, the closely related Hendra virus (HeV) was suspected to persist in *Pteropus* species of fruit bats, leading to the suspicion that pteropid bats may also serve as the NiV reservoir [1]. Subsequent studies have identified NiV RNA and NiV-neutralizing antibodies from urine, saliva, serum, and other organ sites in multiple *Pteropus* bat species throughout Asia as well as in countries without known human NiV infection [21,22,23]. Investigation into risk factors and human exposures revealed a significant association between NiV infection and drinking raw date palm sap, usually collected from December to February, contaminated with the urine and saliva of bats who also drink the sap [24,25]. NiV transmission to humans via date palm sap has been identified as the primary mode of transmission in Bangladesh, although concomitant human-to-human transmission cannot be excluded [10,24].

In 2014, an outbreak of acute encephalitis, meningitis, and severe influenza-like illness in two villages in southern Philippines was attributed to NiV infection and resulted in a case fatality rate of 82% among those with acute encephalitis syndrome [8]. Although some were due to human-to-human transmission, this was the first time that NiV infection was associated with horse slaughter and consumption of horse meat. Moreover, illness and death in horses and other domestic animals that had recently consumed horse meat were subsequently described [8].

In the recent 2018 Kerala, India outbreak, 23 confirmed and probable cases were identified, with transmission occurring in three hospitals, and primary and secondary cases all traced to a single index case [20]. All environmental samples (including from pets and the partially ingested fruits of bats) collected from around the home and workplaces of the index case tested negative for NiV RNA and the source of infection was not confirmed [20].

Although NiV outbreaks have not recurred in Malaysia or Singapore since the initial outbreak in 1998–1999 or in the Philippines since the single outbreak in 2014, nearly annual outbreaks have occurred in Bangladesh with recurrent sporadic outbreaks in India [3,4,20].

## 3. Molecular Biology of the Virus

Nipah virus belongs to the family Paramyxoviridae—the family that also comprises the human pathogenic viruses HeV, measles virus, mumps virus, respiratory syncytial virus, and human parainfluenza virus. Paramyxoviruses possess a single-stranded, non-segmented, negative sense RNA genome fully encapsulated by envelope proteins including a cell receptor binding protein—glycoprotein (G) of henipaviruses, hemagglutinin (H), or hemagglutinin/neuraminidase (HN)—and a separate fusion (F) protein. While cells infected with NiV react strongly with HeV antiserum, cross-neutralization studies have revealed a difference in neutralizing antibodies indicating that NiV and HeV are distinct viruses. Investigations comparing multiple gene regions of NiV to those of other paramyxoviruses have confirmed that HeV and NiV make up a distinct cluster within the *Paramyxoviridae* family, now classified as the *Henipavirus* genus [1].

**Figure 2:**
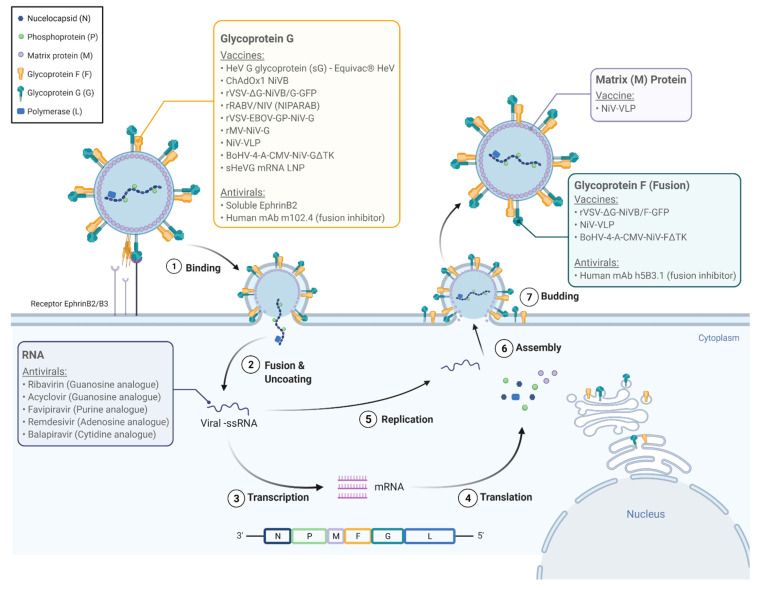
The Nipah virus life cycle and molecular targets for pharmacologic agents. Nipah virus attaches to the EphrinB2/B3 receptor (1) and enters the cell (2). The viral genome is released and is replicated, as well as transcribed into viral messenger RNA, which are in turn translated into viral proteins N (nucleocapsid), P (phosphoprotein), M (matrix protein), G (glycoprotein G), F (glycoprotein F/fusion protein), and L (polymerase). The new viral genome and proteins are assembled, encapsidated, and released from the cell (7). The colored labels highlight the molecular targets for the vaccines and antivirals listed in Tables 2 and 3.

NiV infections are characterized by endothelial syncytium formation, which results in inflammation and hemorrhage. It is postulated that the NiV-G and NiV-F proteins are physically associated and viral fusion results from conformational changes that arise following receptor binding [26]. Guillaume et al. found evidence of a NiV-G protein residue E533 with an important role in receptor binding and similar in structure and function to that of the measles virus attachment hemagglutinin residue R533 [26]. Via 3D modeling of the NiV-G protein, the team identified six other protein residues (W504, E505, N557, Q530, T531, and A532) with what seemed to be important roles in promoting viral fusion as well as in binding to ephrin-B2, a functional receptor for NiV found on epithelial cells and neurons [26,27,28]. Aguilar and colleagues found that the NiV-F protein is glycosylated at multiple sites, reducing fusion efficiency when compared to mutated F proteins and in contrast to N-glycan function in other paramyxoviruses. However, the authors discovered that the N-glycans of NiV may play a role in protecting the protein from neutralizing antibodies [29]. It was also found that the NiV-G and F proteins are not only necessary for binding and fusion to the host cell, but they are also able to facilitate viral budding. However, their function in this process is minor compared to that of the viral matrix protein M which seems to be integral to viral organization and budding [30]. A total of six genes encodes NiV envelope structural proteins (F, G, and M), nucleocapsid protein (N), polymerase (L), and phosphoprotein (P). The P gene encodes phosphoprotein P as well as proteins C, V, and W which play key roles in NiV’s pathogenicity [31]. Figure 2 illustrates the life cycle of NiV within the host cell and potential pharmacologic targets.

The genomes of NiV-B and NiV-M are 91.8% homologous. The NiV-B genome is six nucleotides longer than that of NiV-M, and all six additional nucleotides are found on the 5′ non-translated region of the F protein gene. Among the open reading frames, only the V gene shows variation between the strains [32]. The V gene encodes for a protein (V) with multiple identified mechanisms of suppressing the host immune response in NiV infection, and the significance of the variation in the V gene between NiV strains remains to be elucidated [30,31].

## 4. Pathogenesis and Clinical Features

A study of clinical and autopsy findings of 32 cases of NiV infection during the initial outbreak in Malaysia and Singapore in 1998–1999 found that the mean time from fever onset to hospitalization was 3.3 days, and the mean time from fever onset to death was just 9.5 days. Clinical symptoms of this early outbreak were largely localized to the central nervous system (CNS) and included drowsiness, headache, and some degree of encephalopathy. The most common pathological features were histopathological changes in the blood vessels of multiple organs with evidence of vasculitis of the small vessels. Necrotic plaques were observed in the brain parenchyma of nearly all cases, as well as occasional syncytial or multinucleated giant endothelial cells [1]. Virus isolation from the CSF and IgM detection from the CSF or serum were also common [33].

The strain of NiV responsible for the recurrent outbreaks in Bangladesh (NiV-B) is distinct from the strain isolated in Malaysia and Singapore (NiV-M) and has been associated with different clinical and epidemiological findings [34]. NiV-B is more commonly associated with respiratory symptoms, human-to-human transmission, lack of an intermediary host, and a higher overall case fatality rate of nearly 75% (compared to a rate of 40% related to NiV-M). NiV-B also displays increased intra-strain genetic variability. Although there have not been any large pathological studies done on NiV-B, an animal study using eight African green monkeys found increased lethality and severity of pulmonary histopathology in the NiV-B group compared to the NiV-M group, suggesting viral virulence factors contribute to mortality [34]. 

## 5. Antiviral Therapies in Development

Several antiviral therapies have been investigated for treatment or post-exposure prophylaxis against NiV infection Table 2 and Figure 2. Ribavirin is a guanosine analogue with broad-spectrum antiviral activity against both RNA and DNA viruses, and in vitro activity against both HeV and NiV, inhibiting RNA replication. It was the first antiviral used to treat NiV infection. During the 1998–1999 outbreak in Malaysia, a total of 140 patients with acute NiV infections based on clinical and epidemiological (rather than serological) data were treated with ribavirin, while 54 patients served as controls—chosen primarily due to presentation and treatment prior to the availability of ribavirin. Early in the trial, ribavirin was only available orally with intravenous administration only becoming available later into the outbreak. This initial trial saw a 36% reduction in mortality associated with ribavirin use [35]. However, a second study of the same outbreak saw ribavirin use in 78% of 94 patients admitted with epidemiologically and clinically diagnosed NiV infections without a significant reduction in mortality associated with its use [18]. Ribavirin has not been found to reduce mortality in hamster models when used in combination with chloroquine despite both drugs’ independent efficacy in vitro [36,37]. Ribavirin has also been used as post-exposure prophylaxis in one report of eight healthcare workers in Kerala, India who remained free of NiV disease after receiving ribavirin following exposure to a patient who later tested positive for NiV [37]. In the absence of any proven effective therapy, the National Centre for Disease Control, India recommends ribavirin for NiV infection, but not as post-exposure prophylaxis. During the March 1999 NiV outbreak in Singapore, nine abattoir workers who presented with encephalitis and later tested positive for NiV IgM were empirically treated with ceftriaxone and acyclovir, another guanosine analogue [6]. Among these nine people, eight survived, although whether or not survival was related to acyclovir use remains unclear [6]. Acyclovir studies against NiV in vitro or in additional in vivo trials have not been published. 

Several nucleoside analogues have been shown to inhibit viral replication of NiV. Favipiravir (T-705) is a purine analogue, RNA-dependent RNA polymerase inhibitor with broad-spectrum anti-RNA virus activity, approved in Japan against emerging influenza strains. It has shown activity against henipaviruses in vitro and in a Syrian hamster model [38]. Remdisivir (GS-5734), an adenosine nucleoside analog prodrug with broad spectrum activity against paramyxoviruses, coronaviruses, and filoviruses, has shown improved mortality in non-human primates infected with Ebola virus as well as in vitro activity against NiV [39,40]. The last nucleoside analogue studied against NiV to date is R1429 or 4′-azidocytidine, a cytidine analogue, and its prodrug balapiravir. Balapiravir has shown in vitro activity against both NiV and HeV; however, previous clinical trials for hepatitis C virus and dengue virus demonstrated poor bioavailability and adverse toxic reactions [41]. 

Poly(I)-poly(C_12_U), an interferon inducer, has also shown some efficacy against NiV both in vitro and in a hamster model [37]. Soluble ephrinB2, a functional receptor for NiV G glycoprotein, has been shown to inhibit viral fusion in vitro [28]. Human monoclonal antibody m102.4, designed to target the ephrin-B2 and B3 receptor binding sites of the G glycoprotein, has demonstrated protection as post-exposure prophylaxis after NiV challenge in ferrets and African green monkeys [42,43]. Similarly, human monoclonal antibody h5B3.1 specific to glycoprotein F has shown efficacy against NiV infections in ferrets [44].

## 6. Vaccines in Development

Multiple vaccines have been under investigation based on the increasing knowledge of the molecular biology of NiV (Figure 2 and Table 3). Early studies reported the induction of neutralizing antibodies and protection from fatal infection among mice and hamsters vaccinated with vaccinia virus recombinants expressing either NiV-G or F proteins [26,45]. More recent vaccine studies continue to be based on the G and F proteins. A subunit vaccine incorporating the G glycoprotein of HeV (sharing 83% amino acid identity with the G protein of NiV) showed apparent efficacy in preventing NiV infection in ferrets exposed to lethal doses of NiV. Although viral genome was recovered from two of the five ferrets, none developed any clinical illness [46].

A series of vector-based vaccines are also in development. The ChAdOx1 NiV-B vaccine uses a replication-deficient simian adenovirus vector encoding NiV-B glycoprotein G. Female Golden Syrian hamsters were vaccinated at various intervals prior to inoculation with NiV-B, some followed with a booster dose of vaccine before inoculation. Virus-neutralizing antibodies were isolated from all animals following a single vaccine dose, and all vaccinated animals survived throughout the study without viral RNA isolation from oropharyngeal swabs or on necropsy. In contrast, control animals suffered weight loss, respiratory and/or neurologic symptoms with virus isolated from oropharyngeal swabs and in tissue on necropsy [47]. Identical results were found among animals challenged with NiV-M; however, animals challenged with HeV had poor results [47]. 

**Table 3:** Vaccines in development to prevent Nipah virus infection.

Vaccines	Description	Animal Model	Reference
**Subunit-based**			
HeV-sG (Equivac^®^ HeV)	Subunit vaccine based on soluble HeV G glycoprotein. Elicits cross protective immune response against HeV and NiV. Available for horses in Australia	Ferret	Pallister et al., 2013 [46]
**Vector-based**			
ChAdOx1 NiVB	Recombinant simian adenovirus-based vaccine encoding NiV-B glycoprotein G	Golden Syrian hamster	Van Doremalen et al., 2019 [47]
rVSV-ΔG-NiVB/F-GFP rVSV-ΔG-NiVB/G-GFP	Recombinant vesicular stomatitis virus (VSV) vaccine expressing NiV-B F or G	African green monkey challenge	Mire et al., 2019 [48]
rRABV/NIV (NIPARAB)	Recombinant rabies virus vector expressing NiV G	C57BL/6 mice	Keshwara et al., 2019 [49]
rVSV-EBOV-GP-NiV-G	Recombinant VSV vector expressing Ebola virus glycoprotein and NiV G	African green monkey challenge	Prescott et al., 2015 [50]
rMV-NiV-G	Recombinant measles virus vaccine (rMV) expressing NiV G	African green monkey challenge	Yoneda et al., 2013 [51]
BoHV-4-A-CMV-NiV-GΔTK BoHV-4-A-CMV-NiV-FΔTK	Recombinant bovine herpesvirus vaccine (BoHV) expressing NiV G or NiV F	Pig	Pedrera et al., 2020 [52]
**Virus-like particle-based**			
NiV-VLP vaccine	Purified Nipah virus-like particles G, F, and M proteins	Golden Syrian hamster challenge	Walpita et al., 2017 [53]
**mRNA-based**			
sHeVG mRNA LNP	mRNA vaccine encoding soluble HeV glycoprotein (sHeVG) subunit	Syrian hamster	Lo et al., 2020 [54]

Recombinant vesicular stomatitis virus (rVSV) is a common vector for vaccine delivery and has been used to design a NiV vaccine expressing the NiV G glycoprotein with an incompatible F protein. A total of three African green monkeys challenged with NiV-M intratracheally three weeks after vaccination survived to the end of the study without clinical disease or virus isolation. Notably, two of the three control monkeys developed increased work of breathing and lethargy with oropharyngeal swabs and blood samples positive for NiV RNA, but both recovered and survived to the end of the study [50]. On necropsy, control monkeys displayed histopathologic changes consistent with NiV infection in both brain and lungs whereas vaccinated monkeys showed no changes [50]. 

To address the apparent increased pathogenicity of NiV-B over NiV-M, Mire et al. designed a NiV-B vaccine using rVSV expressing either G or F protein. All vaccinated animals developed NiV-B neutralizing antibodies within three weeks and survived challenge with NiV-B without signs of infection [48]. Recombinant measles vaccine expressing NiV G protein had similar results in African green monkeys, and recombinant bovine herpesvirus vaccine expressing either NiV F or G protein showed protection in pigs [51,52]. 

A live-attenuated rabies virus-based vaccine against NiV was investigated for wildlife to take advantage of the strong humoral immune responses generated by rabies-based vaccines. NiV-B glycoprotein G encoded into the rabies virus vector induced seroconversion in test mice [49]. The authors suggest a live-attenuated rabies-NiV hybrid as a potential vaccine for wildlife with the possibility for dual vaccination against rabies and NiV [49].

Virus-like particles (VLPs) produce strong immunogenicity with both native F and G glycoproteins without the risks associated with using a virion containing viral genome. Walpita et al. demonstrated protection and neutralizing antibody titers among Syrian golden hamsters from a vaccine using NiV VLPs [53]. Lastly, an mRNA vaccine encoding the soluble hendravirus glycoprotein subunit is currently under development, after showing partially protection against NiV infection in Syrian hamsters [54]. 

NiV infections in humans are sporadic, unpredictable, and often self-contained, consequently traditional clinical development pathways are not fruitful. Future studies should focus on using vaccine candidates to prevent NiV infections in exposed individuals on a compassionate basis. 

## 7. Non-Pharmacologic Interventions

Non-pharmacologic interventions have dominated prevention efforts against NiV infection. Initially, this involved the bans on importation of pigs from known endemic regions and the mass culling of pigs and other animals that tested positive or were in close contact to positive animals [1,2,55]. Later prevention strategies to mitigate zoonotic transmission have been aimed at limiting human exposure to date palm sap contaminated with the body fluids of bats. These prevention steps have included boiling date palm sap to molasses rather than consuming it raw and attempting to restrict bat access to date palm trees [56]. Skirts that cover the entire sap collecting system have shown promising results in preventing bats from contaminating the collected sap and have the potential to prevent NiV transmission from bats to humans [12]. 

## 8. Conclusions and Recommendations

While there is strong evidence to suggest *Pteropus* spp. of fruit bats are the natural reservoir hosts for NiV, there is also growing evidence of rapid adaptation of virus to other hosts with varying modes of transmission. In just a few years since it was first discovered, NiV was transmitted to humans via pigs, horses, bats, and other infected humans. While infections due to singular spillover events may be limited to small, isolated outbreaks, repeated spillover events of a pathogen with the potential for human-to-human transmission can result in a much greater disease burden. Although physical barriers to prevent NiV spread between bats and humans may impart some protection, outbreaks continue to occur, and human-to-human transmission remains a threat. Continued research into antiviral drug therapies and vaccines is necessary, as are more comprehensive public health measures comprising a combination of education, hygiene, and animal husbandry practices to prevent potentially larger future outbreaks.

## Figures and Tables

**Table 2:** Summary of drugs with potential antiviral activity against Nipah virus. Legend—mAb: monoclonal antibody.

Drug	Description	Experimental Model	Reference
Chloroquine	4-aminoquinoline	In vitro	Freiberg et al., 2010 [36]
Ribavirin	Guanosine analogue	In vitro	Freiberg et al., 2010 [36]
Acyclovir	Guanosine analogue	Historical review	Paton et al., 1999 [6]
Favipiravir	Purine analogue	In vitro Syrian hamster	Dawes et al., 2018 [38]
Remdesivir (GS-5734)	Adenosine analogue	In vitro African green monkey	Lo et al., 2017 [39] Lo et al., 2019 [40]
Balapiravir (R1479)	Cytidine analogue	In vitro	Hotard et al., 2017 [41]
Poly(I)-poly(C_12_U)	Interferon inducer	In vitro Hamster	Georges-Courbot et al., 2006 [37]
EphrinB2	G glycoprotein fusion inhibitor	In vitro	Negrete et al., 2005 [28]
Human mAb m102.4	G glycoprotein fusion inhibitor	Ferrets African green monkey	Bossart et al., 2009 [42] Geisbert et al., 2014 [43]
Human mAb h5B3.1	F glycoprotein fusion inhibitor	Ferrets	Mire et al., 2020 [44]

## Data Availability

Not applicable.
